# Sliding Scale Theory of Attention and Consciousness/Unconsciousness

**DOI:** 10.3390/bs12020043

**Published:** 2022-02-10

**Authors:** Brad Bowins

**Affiliations:** Centre for Theoretical Research in Psychiatry and Clinical Psychology, Toronto, ON M4S 2C6, Canada; brad.bowins@bellnet.ca

**Keywords:** consciousness, unconsciousness, cognitive unconscious, attention, awareness, neural correlates of consciousness

## Abstract

Attention defined as focusing on a unit of information plays a prominent role in both consciousness and the cognitive unconscious, due to its essential role in information processing. Existing theories of consciousness invariably address the relationship between attention and conscious awareness, ranging from attention is not required to crucial. However, these theories do not adequately or even remotely consider the contribution of attention to the cognitive unconscious. A valid theory of consciousness must also be a robust theory of the cognitive unconscious, a point rarely if ever considered. Current theories also emphasize human perceptual consciousness, primarily visual, despite evidence that consciousness occurs in diverse animal species varying in cognitive capacity, and across many forms of perceptual and thought consciousness. A comprehensive and parsimonious perspective applicable to the diversity of species demonstrating consciousness and the various forms—sliding scale theory of attention and consciousness/unconsciousness—is proposed with relevant research reviewed. Consistent with the continuous organization of natural events, attention occupies a sliding scale in regards to time and space compression. Unconscious attention in the form of the “cognitive unconscious” is time and spaced diffused, whereas conscious attention is tightly time and space compressed to the present moment. Due to the special clarity derived from brief and concentrated signals, the tight time and space compression yields conscious awareness as an emergent property. The present moment enhances the time and space compression of conscious attention, and contributes to an evolutionary explanation of conscious awareness.

## 1. Introduction

The relationship between attention and consciousness has both puzzled and intrigued researchers over many decades. Theories of consciousness invariably address the relationship between these two variables according to Pitts et al. [1] in their review, demonstrating a spectrum from the extremes of attention not being required to absolutely necessary for consciousness:

- Recurrent processing theory (RPT) suggests that consciousness can occur in the absence of attention.
- Higher-order theory (HOT) proposes that sensory perceptions involving attention are insufficient for conscious awareness, and that higher-order processing is required for the conscious experience.
- Attended intermediate-level representation (AIR) theory indicates that phenomenal (experienced) consciousness arises when perceptual representations at intermediate levels of sensory hierarchies are modulated by attention.
- Integrated information theory (IIT) proposes that attention shapes the structure of integrated information (what is in versus out of the major complex), and hence the content we consciously experience.
- Global neuronal workspace theory (GNWT) postulates that attention is necessary for conscious perception.

These theories are largely based on perceptual consciousness, mainly visual research, and human consciousness [1,2,3]. Conscious awareness includes both sensory-perceptual (visual, auditory, olfactory, touch, pain, balance, acceleration, and positioning) and thought forms (focused thoughts, mind wandering, retrieved memories, emotions, problem solving, and self-awareness), plus various combinations, leading Dennett [4] to indicate that consciousness can involve anything and everything. Conscious awareness appears to transpire in animal species varying in cognitive capacity and not just humans with enhanced cognitive ability [5,6,7,8,9,10,11]. According to Fabbro et al [10], there are 5 lines of evidence supporting the presence of consciousness in vertebrate species: first, a pattern of EEG activity in the range of 20–70 Hz, typically linked to wakefulness and REM sleep; second, thalamo-cortical activity; third, widespread brain activity during processing of sensory stimuli; fourth, selective synchronization at cortical and brainstem levels of dynamically formed neural networks involved in binding sensory stimuli; fifth, the presence of egocentric maps for localizing an individual in a given space. Fish, amphibians, reptiles, birds, mammals, and primates, demonstrate some form of consciousness based on these and other indicators of consciousness [6,7,9,10,11]. Furthermore, beyond vertebrates it appears that consciousness occurs in at least more cognitively advanced invertebrates, such as cephalopods including, octopus, squid, and cuttlefish, that rely on decentralized nervous systems in their tentacles to achieve remarkable problem solving feats [12,13], and possibly also arthropods [8]. It has even been proposed that the origins of consciousness might be with the first cellular life forms, prokaryotes, based on subjective awareness [5].

The various theories addressing the link between attention and consciousness, typically rely on more advanced neural structures not present in many species demonstrating conscious awareness. For example, HOT suggests that higher-order processing involving the prefrontal cortex (PFC), and perhaps parietal regions, modifies sensory perceptions to produce awareness, and AIR requires substantial cognitive processing including working memory [1]. Another major problem with higher-order and more advanced neural structures being cited for conscious awareness is that their absence does not eliminate consciousness [2,14,15]. Visual and auditory consciousness have been linked to numerous specific neural cortical and subcortical structures [1,3,14,16,17,18,19,20,21,22,23]. Then there are neural structures associated with olfactory, touch, pain, balance, acceleration, and positioning perceptual consciousness, and also thought-based consciousness encompassing focused thoughts, mind wandering, retrieved memories, emotions, problem solving, and self-awareness [24,25]. Beyond these very specific types, conscious awareness can include combinations, such as the sight and smell of a stimulus, and a sensory experience combined with a thought about it, potentially yielding further neural structures associated with simultaneous forms of conscious experience. It has been noted that research emphasizes the role of a particular neural region in isolation, but each area is imbedded in a network of interacting brain regions [26]. Impairment to the diverse subcortical and cortical structures in isolation or combination, however, does not fully eliminate consciousness, as evident from the spinal cord, cerebellum, amygdala, hippocampus, thalamus, hemisphere commissures, frontal lobes, and prefrontal cortex [2,14,15]. Remarkably, consciousness can still transpire with great quantities of the cortex absent [2]. Hence, these structures with several only present in species with higher cognitive capacity, cannot fully account for conscious awareness [2,14,15].

An additional problem consists of the neural structures, and also connectivity, hypothesized by major theories to play an instrumental role in consciousness, are also utilized by unconscious information processing. The example of emotional information processing demonstrates this occurrence. Emotional information processing involves two crucial components: the detection of core circumstances linked to primary emotions, and from this the generation of the feeling state we identify as the emotional experience. Both unconscious and conscious cognitive activating appraisals detect core circumstances [27,28,29,30,31,32,33,34,35]:

Fear—threat or danger.

Sadness—loss.

Anger—violation or damage.

Disgust—physically or morally repulsive stimuli.

Shame—social or perhaps moral transgression.

Happiness—gain.

Interest—potential reward.

Surprise—unanticipated occurrences either positive or negative.

For the most part, we are unconscious of this process, as evident by how we are unaware of why we experience most of the emotions transpiring in a given day. Conscious thoughts, however, do trigger these emotions, such as, “I know I’ve lost her” for sadness, and “That performance evaluation is going to block my advancement and maybe get me fired” for fear. Part two of emotional information processing—from cognitive activating appraisal to the experienced emotion—is entirely unconscious. For fear alone the amygdala, insula, hippocampus, thalamus, anterior cingulated cortex, and prefrontal cortex are highly involved [36,37,38,39]. Emotion regulation occurs unconsciously relying on connectivity between the subcortical limbic and paralimbic systems and cortical structures, with the prefrontal cortex prominent. Excessive fear involves amplified activity in limbic and paralimbic structures, and decreased activity in the PFC [40,41,42,43]. This pattern is typically interpreted as the PFC failing to exert sufficient top-down regulation of limbic system fear/anxiety responses [41,42,43,44,45,46]. From the example of emotional information processing, it is evident that subcortical and cortical neural structures and connectivity between them, often cited as critical for consciousness, are also instrumental in unconscious information processing—the cognitive unconscious.

Neural connectivity is a crucial component of conscious and unconscious information processing, given that neural structures do not exist and function in isolation. Connectivity plays a significant role in neural correlates of consciousness, defined as patterns of brain activity that specifically accompany a particular conscious experience [16] and minimal neural mechanisms sufficient for any one specific conscious experience [47]. With severely limited or no neural connectivity consciousness is absent. Research evidence indicates how with states of reduced consciousness, such as non-rapid eye movement (non-REM) sleep, anesthesia, coma, vegetative states, epileptic seizures, connectivity appears to be impaired either eliminating or severely restricting conscious awareness [48,49,50,51,52]. Massimini et al. [52] applied transcranial magnetic stimulation (TMS) during non-REM sleep and anesthesia, finding, based on high density-electroencephalogram (EEG), that the initial activation of the thalamocortical system was not sustained, whereas during REM sleep and wakefulness activation persisted allowing propagation of the signal. Focusing just on sleep, Massimini et al. [51] discovered that during quiet wakefulness where there is conscious awareness, TMS stimulation of the premotor cortex spread to connected cortical areas centimeters away after the approximately 15 millisecond initial response, but during non-REM sleep it did not propagate. Sufficient connectivity appears to be necessary to generate information processing [49,50]. Therefore, with severely restricted or no neural connectivity there is an absence of consciousness, related to an absence of information processing. However, this occurrence cannot be cited as only a neural correlate of consciousness: given how extensive information processing also characterizes the cognitive unconscious, such as with emotional information processing, the unconscious mind is also impaired with diminished neural connectivity.

Based on these considerations, a valid theory of consciousness must also be a robust theory of the unconscious mind, a point rarely, if ever, considered. Attention plays a prominent role due to how it is essential for information processing in regards to both the cognitive unconscious and conscious awareness. Furthermore, with consciousness in diverse animal species varying in terms of cognitive capacity and the type of cognition employed, the perspective must be inclusive of the diversity of species and forms of information processing utilized. If it is the case that consciousness transpires in diverse animal species varying in cognition in regards to cognitive capacity and type, consciousness almost certainly evolved [53,54,55]: energy intensive adaptations that characterize diverse species serve an evolutionary fitness enhancing function. Hence, ideally, any valid perspective regarding conscious awareness should provide a coherent explanation for why consciousness evolved. Existing theories of consciousness, mostly focused on human consciousness and that derived from the visual sense, cannot satisfy these criteria. Proposed here is a sliding scale perspective pertaining to the time and space components of attention, and its relationship to unconscious and conscious information processing, the latter yielding conscious awareness. A viable evolutionary explanation drawing on time and space is provided. First, though, a clarification of relevant terms.

Attention is highly relevant to conscious and unconscious information processing, but the definition is elusive: attention can and has been described in various ways, including arousal, alertness, vigilance, sensory detection both spatial and feature, as an executive function linked to working memory, and self-attention [56]. The diversity of perspectives pertaining to what attention actually represents led Hommel et al [57] to claim, “No one knows what attention is.” Despite the difficulties encountered in defining it, I describe it in terms of the most basic element applicable to all forms—focusing on a unit of information. Attention must involve information of some form and that information must be focused on in some fashion. For arousal, there is something that arouses whether an internal or external stimulus comprising information, and for it to arouse it must be detected, which involves focusing. The same applies to alertness, that might be understood more as readiness to focus on a unit of information. Vigilance entails focusing on stimuli to detect something of significance to the organism, and the stimuli comprise information. Both spatial and feature sensory attention involve information that is focused on to be detected and processed. Executive functioning pertains to how information is processed with attention the capacity to focus on a mental or physical task, both involving information. Self-attention requires a focus on some aspect of the self that provides information. Focus is involved in all these variants of attention and information of diverse forms is focused on. Hence, attention as focusing on a unit of information encompasses all possible forms of attention, and every viable type of information.

Relevant to both consciousness and unconsciousness is information processing, and unconsciousness can be understood as either information processing we are not consciously aware of, or an impairment to consciousness information processing due to compromised mental functioning, the latter occurring with little or no neural connectivity for instance. For information processing to transpire at an unconscious level attention must be present, and this is evident with the “cognitive unconscious” [58] encompassing many information capacities, including emotional and non-emotional information processing, executive functions, psychological defense mechanisms, action preparedness, and regulatory control processes, each requiring a focus on various units of information [58,59]. Blind sight, whereby people with damage to the occipital cortex, who are consciously blind, are able to navigate around a space full of objects, also demonstrates attention at an unconscious level [60,61]. There is a debate as to whether blind sight represents genuinely unconscious visual processing or degraded consciousness, and Brogaard [62] presents compelling evidence that it is indeed unconscious information processing.

In contrast to attention which operates in both the unconscious and conscious realms, awareness is restricted to consciousness [63], and the relationship between attention and consciousness is really addressing attention and conscious awareness. Much like attention, awareness is difficult to define, although everyone is aware of having awareness. In a general sense, it is defined as: the mental state of knowing about something [64]; knowledge and understanding that something is happening or exists [65]; knowing that something exists and is important [66]; the ability to know and perceive, feel, or be cognizant of events [67]. In science, awareness is typically used synonymously with consciousness and articles mentioning awareness do not really try and adequately define it, often referring to contents of consciousness [68]. Even though consciousness and awareness are separate terms they appear to be referring to the same occurrence, given that it impossible to be aware and not conscious to some extent, and consciousness without some awareness does not transpire. Naturally, there is a spectrum of consciousness and awareness ranging from minimal to intense, but the two terms are really inseparable. Hence, I will treat them as one and the same, whenever the terms consciousness, awareness, or conscious awareness are applied. Related to the more general definition of awareness, conscious awareness provides the capacity to know about something, with the extent and quality of knowing varying with the cognitive capacity of the organism.

## 2. Sliding Scale Theory

The sliding scale theory of attention and consciousness/unconsciousness describes how attention directly relates to the information processing involved in the cognitive unconscious and conscious awareness. Attention involves focusing on a unit (or units) of information but various along a spectrum of time and space compression. Unconscious information processing is characterized by an extended time frame and diffuse space evidenced by parallel information processing [59,69,70,71]. Regarding the time frame, past elements are represented, such as pertaining to memories, and evolutionary derived processes of numerous types, including but not exhaustive of social cognition, motivation, executive functions and other cognitive capacities, emotional information processing templates, psychological defense mechanisms, and cognitive regulation [59]. In the present moment, emotional and non-emotional information processing utilizing input from diverse body systems transpires. Additionally, anticipated states are compared to encountered states as part of an evolved comparator system [59,70]. Regarding future relevant material, action preparedness occurs involving models and probability estimates of potential events yielding anticipated states [59,70]. With such an extensive range of foci the “space” involved is diffuse covering much ground [59,69,71]. Supporting the diffuse space aspect is how parallel processing is the norm and when complex and multifaceted material needs to be processed in a fairly short time frame, unconscious information processing appears to be superior, due to the parallel function allowing simultaneous analysis of different information streams [69,71]. Visual information processing research demonstrates that attention occurs in the absence of awareness, supporting the application of attention to the unconscious mind [72,73].

In contrast to unconscious information processing, conscious information processing entails attention that is highly time and space compressed. Regarding time compression, conscious attention is restricted to a very brief millisecond to, at most, a few seconds time frame, as revealed by research. Visual perceptual senses respond with feature detection in substantially less than 100 milliseconds [74], and only 100 milliseconds might be required for a sensory stimulus to be included in a conscious scene [75]. Enhancement of the perceptual signal has also been identified around 100 milliseconds [76]. Specialized neural systems for focusing attention on task-relevant target stimuli are engaged at about 200–250 milliseconds [1]. Pertaining to vision, awareness negativity (electrophysiological activity related to awareness) transpires in approximately 200 milliseconds after stimulus onset, and a late positivity signal (event related brain potential) occurs in about 300–400 milliseconds [76,77,78,79,80]. Regarding auditory processing, the awareness negativity and late positivity time frames are the same as for visual processing [18,19]. Conscious events have a cycle of about 100 milliseconds, fading after a few seconds [81]. In general, perceptual conscious awareness transpires in approximately 400 milliseconds [1,82]. Conscious awareness involves brain processes that are very restricted in time [83].

The time frame of conscious attention corresponds to the very brief present moment. It is commonly believed that consciousness is more extensive, such as memories suggesting that conscious awareness extends to the past, but the memory is consciously processed in the present moment only. Future planning appears to extend it beyond the present moment, but the awareness is restricted to thoughts pertaining to the future experienced in the present moment. The “space” of conscious attention is also restricted: given the very brief millisecond to at most a few seconds time frame, attention can only be directed to very limited perceptions or content involving highly condensed space [4,63,75]. Furthermore, we are never aware of the processing that goes into a conscious experience, an occurrence that would involve multiple attentional foci [4]. Conscious experiences are unitary, consistent with a limited attentional focus [75].

Attention that is highly time and space compressed to the present moment gives rise to conscious awareness, derived from the special clarity of brief and concentrated signals. As a thought experiment, imagine a full spectrum of visible light wavelengths representing every hue. This spectrum will appear indistinct fading into the background. Now picture this diffuse image quickly replaced with only one very specific wavelength. Clarity suddenly rushes to the forefront from the time and space (wavelength) compressed light signal. Likewise, imagine every gradient of sound wave in the spectrum of human auditory capacity presented at once. At best, the experience will be of noise that is not distinct and blended with the soundscape. Suddenly, all sound wavelengths except a tight one disappear, yielding a very clear and prominent sound in the forefront. In the realm of thought, all the information processed unconsciously would be experienced as indistinct and overwhelming in the time frame of the present moment, contrasting with the clarity of one prominent thought focus within consciousness. Focused attention and/or a clear signal enhances awareness [84,85,86], supporting the link between time and space compressed attention and conscious awareness. For visual consciousness, the degree of attentional focus and stimulus strength interact to influence consciousness according to research by Pitts et al. [1]: full access conscious awareness occurs when attention and stimulus strength are high. High attentional focus and stimulus strength provide for a clear and salient signal. Pitts et al. [1] suggest that theories of consciousness must account for space, which also implies time considerations given that space and time are linked as space-time.

Hence, attention that is tightly time and space compressed yields conscious awareness as an emergent property. A special clarity emerges from the briefness and concentration of the relevant attentional focus. This process applies to all forms of consciousness, both perceptual and thought, consistent with the notion that conscious experience can include anything and everything [4]. Unconscious information processing, by contrast, does not allow for any awareness due to the time and space diffusion of the attentional foci. Consistent with the sliding scale, it is not either/or, but a gradient of time and space compression accounting for occurrences at the border of consciousness and unconsciousness. This spectrum aligns with how most natural processes are continuous [87,88]. Cognitive events are partially conscious when various perceptions, memory retrieval, emotional states, and related phenomena, are at the margins of the time and space compression yielding conscious awareness. For instance, when driving along a highway, many aspects of the surrounding environment only produce a vague awareness compared to the car ahead or information on the instrument panel. Explicit and implicit attention processing is also relevant, the former when attention is voluntarily focused on goal relevant stimuli, and the latter in response to an inherent feature of a stimulus, such as when novel stimuli appear in the environment or when a picture grabs one’s attention [89]. Explicit attention is focused on relevant (to the organism) information that is time and space compressed, thereby yielding conscious awareness. For example, a person looks for signs of theft upon finding the door to their house forced open. Implicit attention involves some degree of unconscious information processing, that becomes conscious due to the significance. For instance, walking into a department store selling perfumes by the entrance, your attention is suddenly drawn to a particular product. Unconscious information processing evaluated this specific perfume as that worn by an abuser from your past. The emotional significance of the stimuli drew it into the tightly time and space compressed attention of the present moment, yielding conscious awareness. Likewise, some cognitive events are preconscious, mostly processed unconsciously, but capable of being attended to consciously due to the significance. The sliding scale of time and space compression offers a potential way to test the theory. Space, both in regards to actual physical space and objects in the environment, might be systematically varied along with the time frame of information processing from milliseconds to many seconds, with objective and subjective assessments of the clarity of the attention focus. Various senses might be tested in this fashion, including visual, auditory, and olfactory. Although this artificial paradigm cannot match the space and time diffusion of unconscious information processing, the theory predicts that the more time and space compressed the attention focus, the greater clarity, at least to a point, given that very small fractions of a millisecond might be too brief for sensory systems to adequately process the information.

Highly relevant to conscious awareness is its application to the present moment, with a similar time frame of milliseconds to a few seconds. This narrow time frame of the present moment enhances the tight time and, hence, space compression of conscious attention. Additionally, the present moment was likely instrumental in the evolution of conscious awareness shaping the tight time and space compression.

## 3. Evolution of Conscious Awareness

To appreciate the crucial significance of the present moment we must briefly explore the nature of time that has been endlessly debated, major physics theories varying in how they portray it. According to quantum and also Newtonian physics time distinctions are valid, whereas relativity theory does not allow for any distinctions [90,91,92,93,94]. Based on relativity theory, this scenario for time has been interpreted as no-time with past, present, and future distinctions an illusion, and everything present in a landscape of sorts [90]. Such a notion counters the arrow of time and our experience of time passing, the psychological arrow of time with future-present-past. It also counters the presence of time distinctions in quantum physics (see below) and Newtonian physics (cause time A and effect time B). Perhaps the resolution of these disparate perspectives on time resides in the “relative” level of perception: time and space might at a very micro (quantum) level represent stitches yielding a fabric appearing continuous without distinctions at a macro (relativity) level. Much like a uniform garment, if one examines it from a macro vantage point it appears continuous, but if one inspects at a very fine level stitches become evident. The micro quantum stitches might provide for future, present, and past distinctions.

Previously, I proposed that time and, hence, space as space-time have a robust role in the evolution of conscious awareness [95]. Assuming that time distinctions are valid, they are probably based on potentialities, actualization of potentialities, and actualized events. The future consists of potentialities varying in probability. Events that have not yet occurred represent potential occurrences. For example, in the next five minutes there are a wide range of potential occurrences that vary in probability. The present moment is where potentialities are converted into actualized ones. As a thought and practical experiment, attempt to undo what you have just actualized. The actualized event within milliseconds to at most a few seconds is impossible to undo. If too absorbed in texting on your cell phone that you step into the path of a speeding car, this occurrence including the damage cannot be undone. The given actualized occurrence then becomes part of the past, possibly in a record of sorts, supported by the principle of preservation of quantum information [95,96]. The past has occurred and cannot be altered. No process in physics allows for a return to the past [97,98], an event that could alter this component of time.

The notion of future potentialities, present moment actualization of potentialities, and the past actualized potentialities, links well to quantum processes. Within the quantum realm states exist in a superposition [91,93]. When an interaction, such as an experimental measurement, occurs there is collapse of the wave function for the state interacted with [91,92,93,94]. This process is instrumental in the arrow of time, with the future as potential states (the superposition), the present as collapse of the wave function for a given potential state, and then the past as the actualized potentiality possibly in a quantum record. Of course, collapse of the wave function cannot occur at a macro level, such as for the macro movement of a finger, but a compilation of micro quantum wave function collapses yielding the macro form of the finger movement can. The future pertaining to the finger movement consists of a superposition of numerous potential states, and the actualized movement collapse of micro wave functions pertaining to all aspects of that event. The past preserves this quantum wave function collapse in an information record of sorts, that might be referred to as a quantum actualization record [95]. Quantum processes operate within biological systems including at the cellular level [99,100,101,102,103], supporting the possibility of this quantum process for future, present, and past. It has been proposed that quantum collapse of the wave function itself accounts for consciousness [99,100], but with unconscious information processing cognitive potentialities are also being actualized entailing collapse of the wave function, hence the explanation cannot distinguish consciousness. Furthermore, the type of wave function collapse postulated to explain conscious experiences, based on quantum coherent systems, would likely occur too fast with any environmental interaction to influence neural functioning [104].

Regardless of the exact role that quantum processes play, the critical importance of the present moment with the actualization of potentialities, could have driven the evolution of tightly time and space compressed attention yielding conscious awareness. To elaborate, awareness of the present moment is crucial for maximizing the actualization of adaptive potentialities and minimizing the actualization of maladaptive potentialities in this brief time frame [95]. For instance, detecting an unusual movement of nearby grass triggers an immediate realization that a predator might be there, with fear providing motivation to rapidly withdraw. The predator fails to take you or your offspring. The adaptive potentiality of rapidly withdrawing has been actualized, and maladaptive potentialities associated with this event minimized. Likewise, awareness of a car speeding through a red light arrests your forward momentum, thereby averting severe personal injury. On a more positive note, you suddenly become aware of interest from a potential partner and respond with warm eye contact actualizing that adaptive potentiality. It all occurs in the brief present moment, for better or worse, and having awareness of the present moment yields the clarity of information processing, motivation, and rapid responses based on this information processing and motivation, necessary to maximize the actualization of adaptive potentialities and minimize the actualization of maladaptive potentialities in the present moment. In other words, conscious awareness facilitates rapid adaptive shifts in behavior.

It might be suggested that unconscious information processing can suffice instead of conscious awareness, but given the extensive time and space of the cognitive unconscious, the rapid behavioral shifts required in the very brief present moment to maximize the actualization of adaptive potentialities and minimize the actualization of maladaptive potentialities, are unlikely. Furthermore, conscious awareness provides immediate motivation necessary for millisecond shifts in behavior. Even if some information processing relevant to behavioral alterations transpires unconsciously, the products such as fear are conscious, with the awareness providing the necessary motivation for rapid shifts in behavior. Another related critique of the proposed evolutionary mechanism, is that certain fitness relevant behavior that was initially conscious becomes unconscious influencing adaptive responses. For example, physical movements unfolding to prevent a fall were likely conscious, at least to some extent, during the early years of life when postural and action motor programs are expressed and refined, but later become automatic and unconscious, although activated when a fall is imminent. In instances such as this conscious awareness early on has played a role in optimizing adaptive muscle movements to arrest a fall, and with repetition no longer requires full conscious awareness. However, assuming that a person is not highly intoxicated or medically compromised as with a seizure, a fall always involves conscious awareness, and often adaptive behaviors for the particular circumstance, such as awareness of a nearby object that can be grabbed to arrest the fall. The limitations of information processing without conscious awareness becomes evident in species where very straightforward stimulus-behavior linkages have evolved directing responses. For example, moths have evolved to move towards light, possibly due to nocturnal movement being guided by the moon, and hence light automatically triggers movement towards the source. The attraction of moths to flames reveals the extreme adaptive limitations of this automatic stimulus-response linkage. Additionally, for the vast majority of organisms demonstrating some degree and form of consciousness, behavior required for adaptive responses is far too complex for these automatic stimulus-behavior linkages to suffice. It is likely that once organisms with more extensive cognitive capacities evolved, such that automatic stimulus-behavior linkages could not possibly suffice, conscious awareness also evolved providing for millisecond to second alterations in behavior to maximize the actualization of adaptive outcomes and minimize the actualization of maladaptive outcomes.

Relevant to this postulated evolutionary process is how conscious awareness appears to transpire in animal species varying in cognitive capacity and not just humans with enhanced cognitive ability [5,6,7,8,9,10,11]. It then is not the presence of conscious awareness that varies but the quality based on cognitive capacity. Organisms of more limited cognitive capacity rely on perceptual consciousness, with thought-based consciousness in more cognitively advanced species. Self-awareness appears to be the most complex and relegated to species passing the mirror-test involving recognition of self in a mirror [105]. Within a given organism, self-awareness might initially develop from distinguishing self from objects including other beings in the moment, and as development proceeds identifying the characteristic ways of consciously experiencing things and self within the time-space continuum [106]. Self-awareness can optimize adaptive responses, such as when a person is self-aware that mobility is an issue due to hip or knee problems, and compensating proactively to prevent a fall. By shifting behavior from reactive to proactive self-awareness adds adaptive value. Hence, conscious awareness is a feature of many life forms necessitating an explanation of how it actually materializes applicable to the diversity represented, and not just more cognitively advanced species such as humans. The evolution of tight time and space compressed attention to the present moment yielding conscious awareness, provides a cross-species mechanism, and is consistent with how attention varies in regards to time and space diffusion.

## 4. Discussion

Major theories of consciousness (conscious awareness) focus on human perceptual consciousness, in regards to either or both the content of the theory or experimental paradigms cited to support it. Recurrent processing theory postulates that consciousness arises with recurrent processing in sensory systems involving interconnected feedforward and feedback connections, but without the involvement of widespread areas [107,108]. Global neuronal workspace theory relies on widespread neural connections, consciousness transpiring when a state (content) is present in the neural workspace making it accessible to multiple systems [109,110]. Attended intermediate-level representation theory indicates that perceptual processing at intermediate levels of sensory hierarchies is required [111]. Higher order theories propose that consciousness awareness of a state occurs when a one represents oneself in that state, with higher cortical functioning required [112]. Information integration theory indicates that consciousness derives from effective information transpiring when the integration involving connected nodes is greater than the sum of the information from the separate nodes [113].

When the full spectrum of conscious awareness is considered, including various perceptual forms of consciousness and thought variants, and the diversity of species demonstrating consciousness, these theories falter given that they rely on perceptual consciousness mostly visual, and primarily human consciousness [1,2,26]. Problems pertaining to specific theories transpire. For example, RPT relies on sensory systems which excludes thought forms of consciousness and combined forms. GNWT depends upon access to a global neural workspace that might not apply to numerous species demonstrating consciousness. AIR focuses on perceptual processing at intermediate levels of sensory hierarchies, limiting higher level thought forms of consciousness. HOT requires higher cortical processing, and certainly the PFC, but it appears that consciousness can transpire without these structures [2,14,15]. IIT emphasizes the effective information value between nodes, but unconscious information processing also involves connectedness, such that the information value is greater than that of the independent structures. Furthermore, none of the theories explains why the circumstances postulated actually produce the conscious experience: even if consciousness involves recurrent processing in sensory systems, a state present in a neural workspace making it accessible to multiple systems, employs intermediate levels of perceptual processing, involves one representing themselves in a state with higher cortical processing, or entails effective information from connected regions, why does this produce conscious awareness? There is no clear reason why conscious awareness emerges. Nor is any evolutionary account of conscious awareness provided.

An important consideration pertaining to conscious awareness is attention and each of the theories refers to this, although with varying degrees of reliance (see the Introduction). Of crucial significance, is attention restricted to consciousness or applicable to the unconscious mind? The sliding scale theory postulates that attention plays a key role in both the unconscious and conscious mind: information processing applies equally to the cognitive unconscious and consciousness, and attention is required for information processing. Attention varies along a sliding scale of time and space compression, with unconscious attention characterized by diffuse time and space. Conscious attention is very tightly time and space compressed, yielding awareness as an emergent property derived from the special clarity of brief and concentrated signals. Enhancing the tight time, and hence space, compression is the application of conscious attention to the very brief present moment. Given how the present moment is pivotal for evolutionary fitness due to potential occurrences being actualized in that very brief time frame, the influence of the present moment appears to have driven the evolution of tightly time and space compressed attention. Awareness of the present moment maximizes the actualization of adaptive potentialities and minimizes the actualization of maladaptive potentialities, derived from the clarity of information processing, motivation, and rapid responses based on this information processing and motivation. Conscious awareness essentially facilitates rapid adaptive shifts in behavior. The proposed mechanism for conscious awareness explains its occurrence across all forms of perceptual and thought consciousness, and for species varying in cognitive capacity.

## Data Availability

Not applicable.
